# A Boolean probabilistic model of metabolic adaptation to oxygen in relation to iron homeostasis and oxidative stress

**DOI:** 10.1186/1752-0509-5-51

**Published:** 2011-04-13

**Authors:** Fiona Achcar, Jean-Michel Camadro, Denis Mestivier

**Affiliations:** 1 Modelling in Integrative Biology, Institut Jacques Monod - UMR7592 - CNRS - Univ. Paris-Diderot, Paris, France; 2 Mitochondria, Metals and Oxidative Stress, Institut Jacques Monod - UMR7592 - CNRS - Univ. Paris-Diderot, Paris, France

## Abstract

**Background:**

In aerobically grown cells, iron homeostasis and oxidative stress are tightly linked processes implicated in a growing number of diseases. The deregulation of iron homeostasis due to gene defects or environmental stresses leads to a wide range of diseases with consequences for cellular metabolism that remain poorly understood. The modelling of iron homeostasis in relation to the main features of metabolism, energy production and oxidative stress may provide new clues to the ways in which changes in biological processes in a normal cell lead to disease.

**Results:**

Using a methodology based on probabilistic Boolean modelling, we constructed the first model of yeast iron homeostasis including oxygen-related reactions in the frame of central metabolism. The resulting model of 642 elements and 1007 reactions was validated by comparing simulations with a large body of experimental results (147 phenotypes and 11 metabolic flux experiments). We removed every gene, thus generating *in silico *mutants. The simulations of the different mutants gave rise to a remarkably accurate qualitative description of most of the experimental phenotype (overall consistency > 91.5%). A second validation involved analysing the anaerobiosis to aerobiosis transition. Therefore, we compared the simulations of our model with different levels of oxygen to experimental metabolic flux data. The simulations reproducted accurately ten out of the eleven metabolic fluxes. We show here that our probabilistic Boolean modelling strategy provides a useful description of the dynamics of a complex biological system. A clustering analysis of the simulations of all *in silico *mutations led to the identification of clear phenotypic profiles, thus providing new insights into some metabolic response to stress conditions. Finally, the model was also used to explore several new hypothesis in order to better understand some unexpected phenotypes in given mutants.

**Conclusions:**

All these results show that this model, and the underlying modelling strategy, are powerful tools for improving our understanding of complex biological problems.

## Background

A large body of data suggests that mitochondrial abnormalities may link gene defects and/or environmental challenges to many pathologies including several neurodegenerative processes (for reviews, see [1-4]). Mitochondria are essential organelles serving as the main site of oxygen use within cells. The divalent reduction of oxygen by the respiratory chain is tightly coupled to ATP synthesis by the oxidative phosphorylation machinery. However, a small proportion of the electrons passing through the electron transport chain reacts with molecular oxygen in a monovalent reduction reaction [5]. This process yields the superoxide anion, which can be converted into other reactive oxygen species (ROS), such as hydrogen peroxide and the highly reactive hydroxyl radical, through enzymatic and non-enzymatic reactions [6]. Cells possess an impressive arsenal of weapons for counteracting excess ROS production, including superoxide dismutases, catalases, peroxidases and low molecular mass redox compounds, such as ascorbic acid and glutathione. However, overproduction of the superoxide anion due to the abnormal reduction of key components of the respiratory chain (e.g. ubiquinone and cytochrome bc1) or to the impairment of antioxidant defences adversely affects various cellular processes and constituents (for recent reviews see [7-9]). Disruptions of the respiratory chain or cellular defences are thus increasingly being implicated in acquired and inherited diseases and appear to play a key role in the aetiology of many neurodegenerative disorders, including Alzheimer's and Parkinson's diseases [10,11].

Due to its unique redox properties and chemical reactivity, iron appears to be a key player in abnormal ROS generation, principally as a catalyst of the Fenton and Haber-Weiss reactions [12]. This essential micronutrient is the redox component of the haem and iron-sulphur cluster [FeS] cofactors of many important proteins or enzymes. Iron homeostasis is thus tightly regulated, at all levels. The deregulation of iron homeostasis due to gene defects or environmental stresses leads to a wide range of diseases, from anaemia (iron deficiency) to haemochromatosis (iron overload) [13-15] with consequences for cellular metabolism that remain poorly understood. The modelling of iron homeostasis in relation to the main features of metabolism, energy production and oxidative stress may provide new clues to the ways in which changes in biological processes in a normal cell lead to disease.

In the growing field of systems biology, several attempts have been made to model cellular processes. For complex systems, these models can be classified into static [16,17] and dynamic models [18-20]. Dynamic models are generally analyzed and/or simulated with specific methods as a function of the availability (or lack) of numerical data. The approaches used include ordinary differential equations, stochastic simulation algorithms [21-23], agent or rule-based approaches [24,25], Boolean approaches (probabilistic or otherwise) [26-29], or Petri nets (with hybrid extensions) [30-32]. Most of these models are based on well known pathways (cell cycle control, glycolysis, signal transduction, etc.), or well studied processes relevant to cell physiology (e.g. action potential propagation in neurons).

Our understanding of the relations between oxidative metabolism and iron homeostasis is based on a large body of qualitative knowledge from heterogeneous sources, often lacking numerical data. It is therefore not possible to derive mathematical relationships based on biological knowledge for the entire system, and the model has to include uncertain knowledge. As a consequence, despite several attempts to construct models accurately describing certain aspects of iron homeostasis (at the level of the cell [33] or organism [30,34,35]), no formal model linking iron homeostasis to metabolism control and oxidative stress has yet been developed.

We describe here an approach based on probabilistic Boolean modelling that can deal effectively with vast amounts of heterogeneous knowledge not always associated with quantitative data. Using this approach, we were able to construct a realistic model of cell fate including oxygen, carbon, nitrogen, sulphur, phosphate and iron homeostasis. This methodology dealt well with the mixture of precise and uncertain knowledge. Despite its large size (642 elements and 1007 reactions), we were able to simulate the model and analyse its dynamics. We focused on a simple unicellular eukaryotic system, the yeast *Saccharomyces cerevisiae*, which has many features (genes, proteins, pathways, cell compartmentalisation) similar to those of mammalian cells. We first validated the model by simulating 198 *in silico *mutations resulting from the deletion of individual genes from the model (with a small number of elements kept constant in the model). An independent validation was provided by analysing the key transition from anaerobic to aerobic metabolism, by comparing *in silico *reactions frequencies with experimental fluxomic data [36]. The results of individual deletions were compared with experimental data for real mutants, for which detailed phenotypic analyses were available. We were able to classify the *in silico *mutants into groups of similar "phenotype" profiles, making it possible to identify original properties of the model. The model was used to explore several alternative hypothesis in order to better understand some unexpected phenotypes in mutants.

In this study, we focused on iron homeostasis and oxidative stress. However, we believe that the proposed modelling strategy could be useful for other systems. Typically, it should allow the building of large models with a high level of biological relevance that can cope with both a lack of numerical data and precise knowledge.

## Results and Discussion

### The model

#### Choice of the type of model

In eukaryotic cells grown under aerobic conditions, energy production may depend strongly on oxidative phosphorylation. The efficiency of this process is dependent on tight coupling between the production of reduced equivalents (mainly NADH and FADH2) through interconnected metabolic reactions and their reoxidation by the complexes of the respiratory chain. The reoxidation steps generate the electrochemical gradient required to drive ATP synthesis. The assembly of the respiratory complexes requires the coordinated synthesis of the protein components and their appropriate cofactors, mainly haem or iron-sulphur clusters. Any attempt to model these processes and their relationships must therefore include a description of the main metabolic pathways, ion transport and assimilation, the expression and regulation of genes, protein complex assembly and cofactor biosynthesis. These different levels of description involve several cellular compartments, such as the cytosol, mitochondria and nucleus. Yeast is a particularly suitable cell model for studies of this type, as most of its biochemical pathways have been characterised, from enzymatic reactions to gene regulation. However, some processes, such as the assembly of iron-sulphur clusters, still contain reactions about which we know little (for reviews, see [37,38]). Thus, any model developed to analyse the relationship between iron homeostasis and oxidative stress damage must be able to deal with several different levels of knowledge about a large number of elements (i.e. biological species: proteins, genes, ions, metabolites...) and reactions. It should also include different levels of regulation in different cellular compartments. Furthermore, we wanted to model cellular processes as closely to the molecular mechanisms as possible. For example, if a protein is absent in some specific condition we could have modelled this fact using a negative statement. Instead, we would rather model the underlying molecular mechanism that are easily transcribed using only positive statements. In our example, it means describing why this protein is absent: is the protein degraded in this specific condition? Is the promoter of the corresponding gene occupied by some transcription factor? Is the mRNA degraded?

Our modelling approach is derived from the Biocham language [39], a rule-based formalism. Each biological species (elements) in the model is either "ON" (present) or "OFF" (absent). The elements interact through biological reactions, formalised as rules. We have extended the Biocham approach by adding a weighting to each reaction, making it possible to define a probability for each reaction to simulate the large differences encountered between certain reaction rates (see Methods). This Boolean probabilist approach is designed to mimic the biological reality as closely as possible, given the qualitative nature of the model.

#### The list of elements and reactions

**Figure 1:**
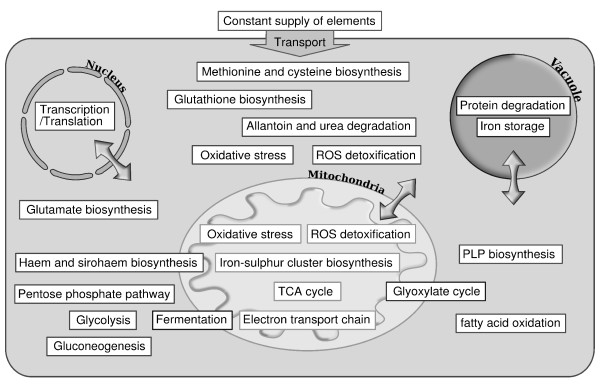
**Overview of the content of the model**. Main pathways included in the model and their cellular localisation.

**Figure 2:**
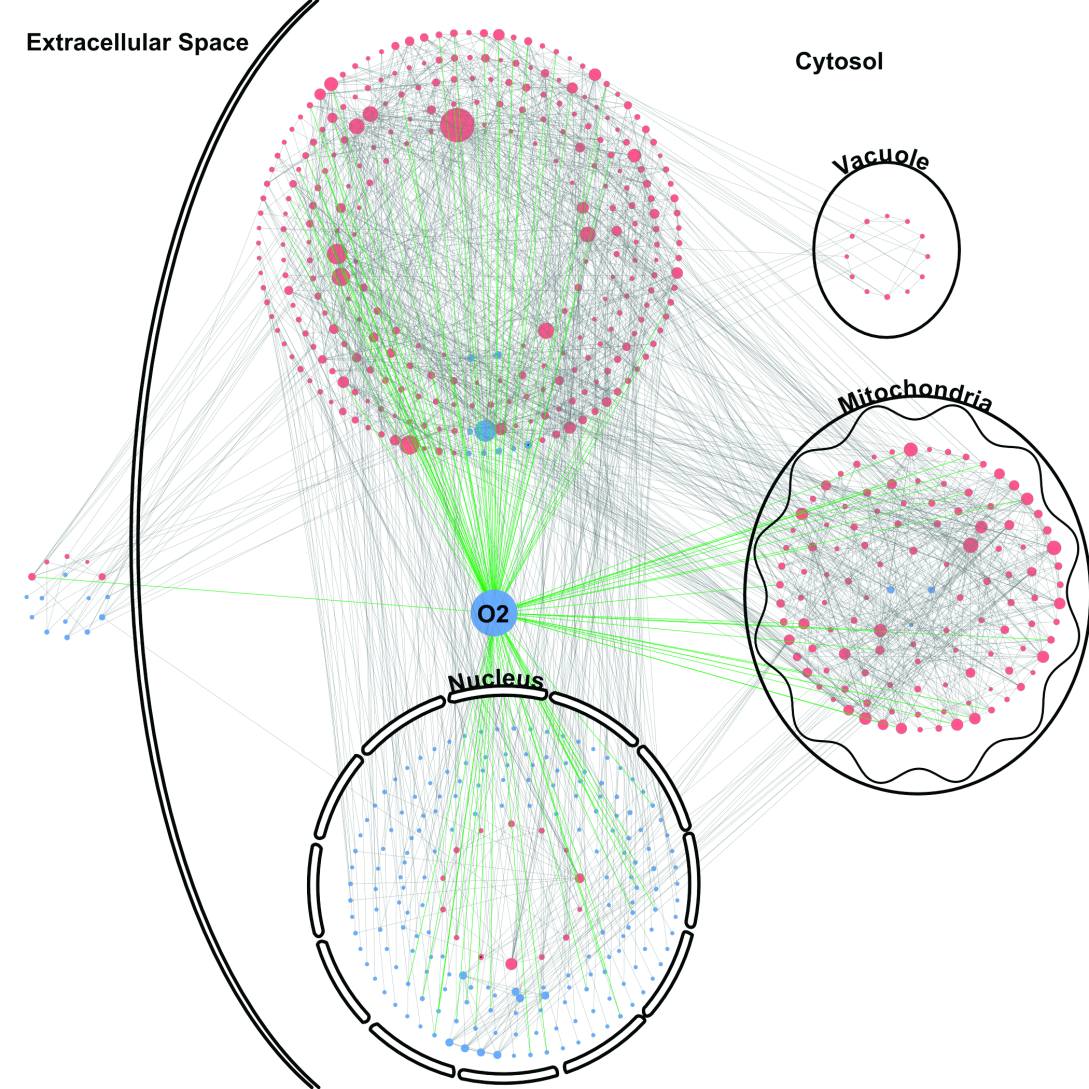
**Graph of the model elements**. Each element is a node, two elements are connected if they are involved in the same reaction. Blue nodes are constant elements, red nodes are non-constant elements. Green lines are the connections involving oxygen. The node size increases with the number of elements connected to it.

The model was built from a core of reactions involved in iron homeostasis. We included in the model the main biochemical pathways, carbon, nitrogen, sulphur and phosphate metabolism, taking into account the cellular distribution of the components and the reactions (see Methods and Figure 1). The connectivity between reactions is depicted in Figure 2. The extracellular space contains the nutrients imported into the cell when the appropriate permeases or uptake systems are synthesized. The nutrients include glucose as a source of carbon. Its uptake may be modified by assigning different weights to the import reaction. When transcribed, the nuclear genes produce proteins that are exported to the cytoplasm and then targeted to their final subcellular compartment (transcription and translation are modelled as one reaction because no alteration of the mRNAs, for example active degradations, was introduced). Mitochondria perform the main energy-related metabolic reactions. Substrates and products shuttle between cytoplasm and mitochondria through appropriate translocators. A limited number of functions were attributed to the vacuole, mostly relating to the homeostasis of metals and phosphate. Our model currently includes 642 elements and 1007 reactions (see Table 1 and Additional file 1 for the detailed list of reactions). The weights of the reactions were set to the default weight of 1 unless changing the weight of a reaction was necessary to model a phenomenon realistically (see Methods for more details and Table 2).

**Table 1:** Number of elements and reactions of the model

642 Elements:	
188	Genes
304	Proteins and protein complexes
125	Other chemical components and metabolites
25	Unlimited elements (always ON)

**1007 Reactions:**	
233	Gene transcription/translation
87	Translocation of elements between compartments
40	Protein complex synthesis/cofactor binding
18	Sulphur metabolism/glutathione biosynthesis
13	Haem/siroheme/FeS/PLP synthesis
67	ATP/GTP synthesis
36	Oxidative stress
419	Element degradation
5	NADP/NADPH synthesis
5	Allantoin (NH3)

**Table 2:** List of the reactions with weights other than the default value of one.

Reactions	Weight	Source and/or comment
Basal transcription/translation of condition or transcription factor dependent genes	0.01	Should be significantly lower than regulated expression

Active Degradation	5	Should be significantly higher than the production

Aft1/2p::nucleus = [Glt1/Aco1p-FeS::c, Grx3p::nucleus] ⇒ Aft1/2p::c	10	[73]

Low affinity Fet4p metal transport	0.1	[74]

Glutathione/Cysteine mitochondrial import	0.1	Balance of flux between compartments in wt

Neoglucogenesis enzymatic reactions	0.001	[75]

Reaction catalysed by Zwf1p	10	Major source of NADPH. [76]

TCA cycle (reverse)	0.1	[77]

Atp production by Atp-synthase	5	[78]

O2m::c = [Sod1p-Cu-Zn::c] ⇒ H2O2::c	100	[79]

O2m::m = [Sod2p::m] ⇒ H2O2::m	100	[79]

O2m::(m or c) + H2O2::(m or c) ⇒ Ot::(m or c)	0.2	[79]

O2m::(m or c) + H::(m or c) ⇒ H2O2::(m or c)	0.1	[79]

Ot::(m or c) + Aco1p-FeS::(m or c) ⇒ Aco1p::(m or c)	0.1	Less probable than aconitase reaction but more probable than spontaneous degradation.

O2m::(m or c) + Aco1p-FeS::(m or c ⇒ Aco1p::(m or c)	0.1	Same as "Ot::(m or c) + Aco1p-FeS::(m or c)".

Ot::(m or c) + any iron-sulphur cluster containing protein ⇒ protein without the iron-sulphur cluster	0.05	Less probable than the same reaction involving Aco1p-FeS

O2m::(m or c) + any iron-sulphur cluster containing protein ⇒ protein without the iron-sulphur cluster	0.05	Less probable than the same reaction involving Aco1p-FeS

Ot::c + Met5p-siroheme-FeS::c ⇒ Met5p-siroheme::c	0.01	Less probable than other FeS proteinsdue to presence of siroheme

O2m::c + Met5p-siroheme-FeS::c ⇒ Met5p-siroheme::c	0.01	Less probable than other FeS proteins due to presence of siroheme

H2O2::c = [Ctt1p-Heme::c] ⇒ O2	10	Higher turnover number than most enzymes but less than SODs [80]

H2O2::m = [Cta1p-Heme::m] ⇒ O2	10	Higher turnover number than most enzymes but less than SODs [80]

#O2::ext ⇒ #O2::ext + O2	10	Provides saturating oxygen.

#glucose::ext ⇒ #glucose::ext + glucose::c	0.1	Limited glucose uptake as described in [36]

Degradation of protein or peptide elements	0.01	Must be significantly less than the default.

Degradation of other elements	0.001	Less than proteins degradation.

m or c = mitochondria or cytoplasm.

#### Simulations

The output of one simulation is "ON" (present) or "OFF" (absent) for each 642 element at each iteration (simulations of 20 millions of iterations or until a steady state was reached, See Simulations in the Methods section for more details). In order to describe the average evolution of each element, each model was simulated 100 times. We then defined, for each element, a parameter called PoP (Percentage of Presence). The PoP is the number of iterations where the element is "ON" during the last million of iterations, averaged over 100 simulations. For example, a PoP for the element NADPH::cytoplasm of 24.3% means that, on average, during the last millions of iterations, this element is "ON" for 24.3% of the iterations. Recall that the model is a Boolean one, and one should not try to interpret or relate these values to quantities such as concentration or number of molecules. However, because an element can be involved into several reactions (and consequently consumed when one of these reactions is triggered), this parameter reveal the "availability" and the "usability" of the element during the time evolution of the system. Hence, it is a simple parameter that convey important biological information.

We also defined a reference model, referred to as the wild type model (WT model) which correspond to a model where all genes are "ON" during the simulations. In this WT model, our list of reactions and their weights allow the PoPs to be: 1/biologically meaningful according the data from the literature and, 2/high enough so that no reaction is always impossible during WT simulations. However some elements, such as the ROS, should remain low in the WT model (PoP less than 1%) as observed in wild type cells. A higher PoP for these elements would be interpreted as related to a situation of oxidative stress. To reach this goal, in some specific cases, the default weights should be altered. As an example, when every weights in the WT model are set to 1, the PoP of the hydroxyl radical (the most deleterious ROS) is higher than 70%. It means that this element would be too often encountered in the system which is obviously biologically irrelevant. In our WT model, this element has a PoP lower than 1% due to appropriate weights on the reactions involved in handling ROS (see Table 2 for a list).

In the following, we used the PoP to compare the outputs of one model (for example when simulating an *in silico *mutation) versus the reference model (WT model).

**Figure 3:**
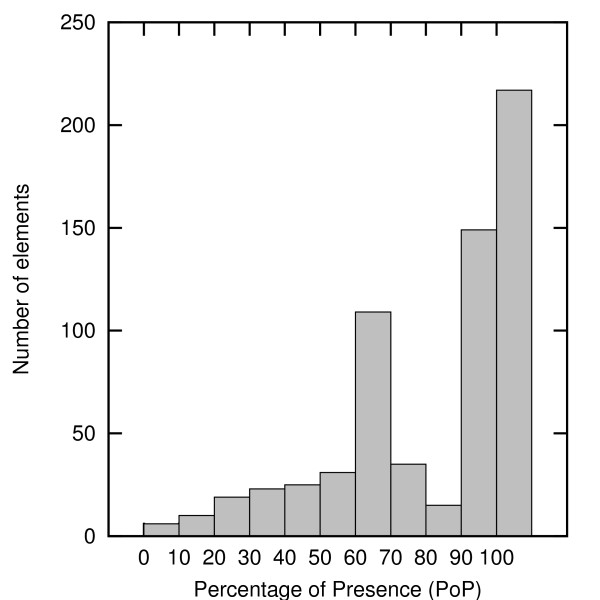
**Histogram of the PoPs of the elements in the WT model at steady state**.

The distribution of the PoP for the various elements (Figure 3) indicated that the vast majority of elements remained present at steady-state levels from a few percent (e.g. toxic forms of oxygen) to 100% (e.g. all the genes). The PoP of most proteins was relatively high (higher than 50%), given their low probability of degradation. Eighty proteins are active in a specific cellular compartment other than the cytoplasm. Since they are produced in the cytoplasm and then translocated to their compartment, the overall PoP for their residence in the cytoplasm is about 66%. This causes the peak around 60% in Figure 3.

We performed a sensitivity analysis of the outputs of the model (PoP at steady state) when the weights of the reactions were modified by multiplications or divisions up to a factor of 10 (see Methods and Additional file 2 for more details). Varying the weight of a given reaction had no impact on the model when the variation was small (weight +/- 10%). For some reactions, when the variations were larger, the PoP of few elements (up to 20 out of 642) was modified. Each of these variations has only a local impact on the graph (as defined in Figure 2). This analysis shows that the model is robust to modifications of the weights of the reactions within a dynamic range of 100, that reflects most biological situations.

### Validation of the model

Our validation strategy involved evaluating the consistency of simulations of the model at different levels. We first considered the effects of removing every gene, thus generating an *in silico *mutant. The simulations of the mutants were compared to experimental phenotypes of the corresponding biological mutants. A second validation involved analysing the anaerobiosis to aerobiosis transition. Therefore, we compared the simulations of our model with and without oxygen to experimental metabolic flux data.

#### Evolution of representative elements in selected mutants

For validation of the model, we compared the results of the simulations for the WT model (all elements present at the beginning of the simulations) with those for models in which specific changes were introduced.

We analyzed the effects of removing selected genes, thus generating *in silico *mutants. The resulting mutated genes have the same designations as the corresponding biological mutants, but with an "is-" prefix (e.g. is-hem15 represents the model with the HEM15 gene switched "OFF" during the simulations - see Methods for more details).

**Figure 4:**
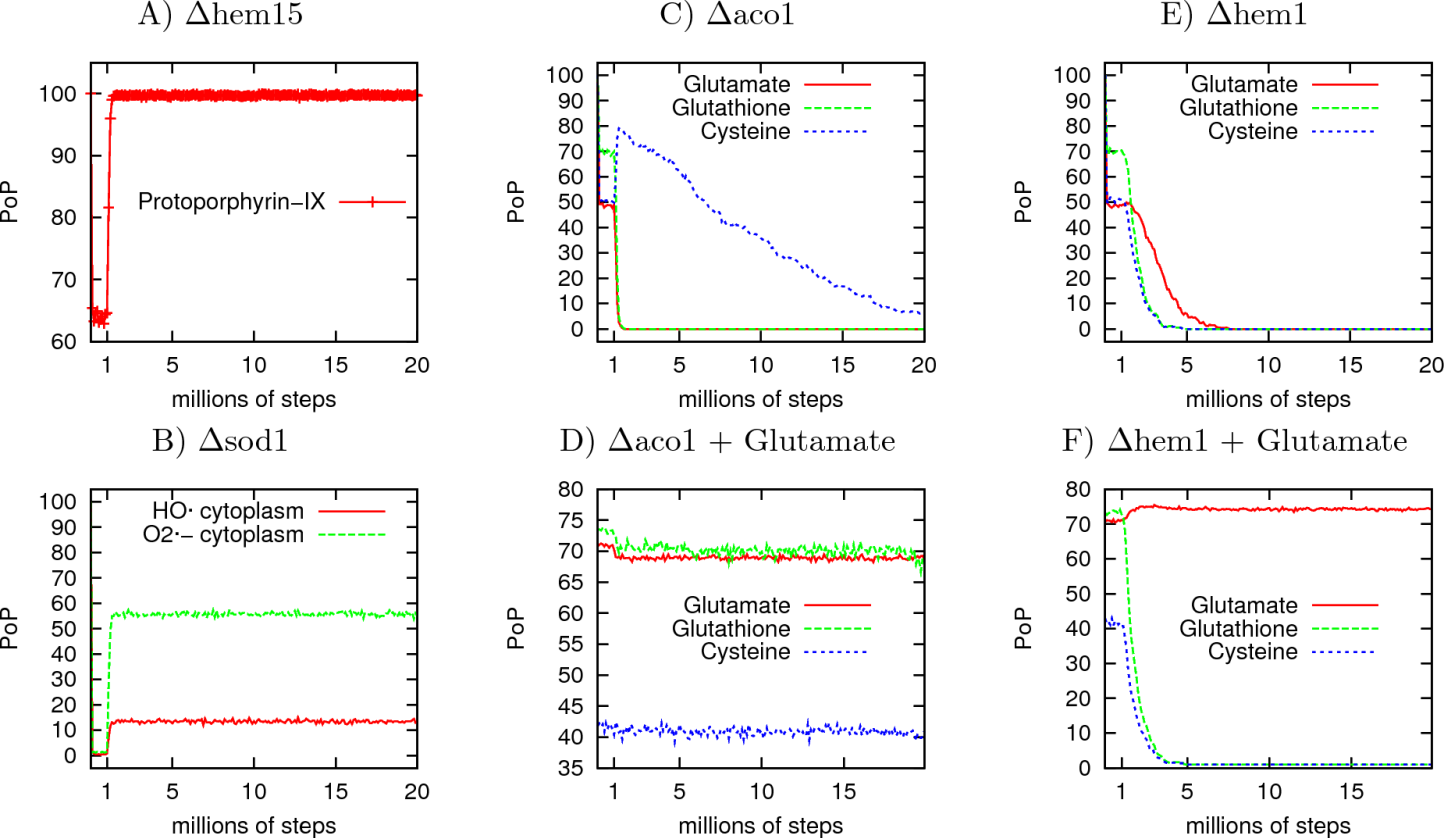
**Mean PoP of a number of elements in selected mutants**. The gene was turned o after the first million of steps. A: PoP of protoporphyrin-IX when the gene HEM15 is turned o, B: superoxide anion and hydroxyl radical when the gene SOD1 is set to "OFF", C-F: PoP of glutamate, cysteine and glutathion C) when ACO1 is set to "OFF". Cysteine reached zero at steady-state, D: ACO1 is set to "OFF" and a source of glutamate is added, E: HEM1 is set to "OFF", F: HEM1 is set to "OFF" and a source of glutamate is added.

We show the evolution of the PoP during the simulations for selected elements in representative mutants in Figure 4. Figure 4-A describes a simple situation in which deletion of the gene encoding ferrochelatase (is-hem15) leads to an increase in the PoP of protoporphyrin IX, the substrate of the enzyme, paralleling the situation occurring *in vivo *in the corresponding biological mutant [40]. Similarly, in a model in which superoxide dismutase was eliminated (is-sod1, Figure 4-B), the PoP of its substrate, the superoxide anion [41] increased strongly. As a consequence, the PoP of the hydroxyl radical also increased, due to the Fenton and Haber-Weiss reactions.

Figure 4-C-F illustrates two more complex biological situations. Yeast mutants lacking the tricarboxylic acid cycle enzyme aconitase are known to be auxotrophic for glutamate [42], due to a lack of alpha-ketoglutarate and impaired nitrogen assimilation. The is-aco1 mutant (Figure 4-C) displayed a decrease in the PoP of glutamate and, as a consequence, a decrease in the PoP of glutathione (GSH), the synthesis of which requires glutamate (GSH is a γ-glutamyl-cysteinyl-glycine tripeptide). However, the PoP of cysteine was not affected in the is-aco1 mutant. Indeed, it may even have increased slightly, as cysteine was no longer used for GSH synthesis. If we "supplemented" our model with glutamate (Figure 4-D), fixing this element as "always present", then the is-aco1 mutant no longer displayed defective GSH synthesis. Similarly, mutants affected in the early steps of the haem biosynthesis pathway are known to be auxotrophic for cysteine/methionine, due to a defect in the synthesis of siroheme, the cofactor of sulphite reductase [43]. Therefore they display impaired sulphur assimilation. An analysis of simulations for the is-hem1 mutant (Figure 4-E) showed a decrease in the PoP of cysteine when hem1 was removed from the model. Accordingly, the PoP of GSH also decreased if siroheme synthesis was impaired. More surprisingly, the model predicted a decreased in the PoP of glutamate for the is-hem1 mutant. The provision of unlimited amounts of glutamate did not restore cysteine and glutathione synthesis in the is-hem1 model (Figure 4-F). Furthermore, glutamate synthesis was restored by providing cysteine as an unlimited element in the is-hem1 model. The decrease in the PoP of glutamate in the is-hem1 model is therefore a consequence of the decrease in the amount of cysteine. Indeed, Figure 4-E shows that the PoP of cysteine decreases before the PoP of glutamate. (for more analysis of phenotypes, see "Systematic analysis of predicted mutant phenotypes" below). The "phenotypes" of such mutants were found to be consistent with published data for the phenotypes of the corresponding biological mutants, providing the first demonstration that our model was of high quality.

#### Systematic analysis of predicted mutant phenotypes

We simulated the model in which each element (gene or element supplied as unlimited) was removed individually from the model (100 simulations for each of the 198 elements removed). Whenever possible, we compared the is-phenotypes to the manually curated phenotypes reported in the *Saccharomyces *Genome Database (SGD). We extracted from the simulations and from SGD, data corresponding to documented phenotypes related to "auxotrophies", "chemical compound accumulation", "oxidative stress resistance", "respiratory growth" and "nitrogen source utilization" (see Additional file 3). The simulations of the different is-mutants gave rise to a remarkably accurate qualitative description of most of the experimental phenotype. There are some unavoidable discrepancies that may be traced back to different problems.

The "auxotrophies" phenotypes were found consistent in 18/21 simulated mutants. We did not evidence a methionine auxotrophy in the is-zwf1 mutant, because in the corresponding model, the decrease in PoP of NADPH is not important enough (PoP of NADPH::cytoplasm: WT = 24.3+/-0.3%, is-zwf1 = 17.7+/-0.3%). NADPH remains present due to the NADH kinase activity and a constant supply in NAD in the model.

We analysed the sensitivity of the is-mutants to oxidative stress (SGD "oxidative stress resistance"). Under our simulation conditions, the mutants were not challenged with large amounts of exogenous hydrogen peroxide, paraquat or diamide as oxidizing agents (all these conditions are usually required to evidence *in vivo *sensitivity to oxidative stress). Therefore, the simulations are not expected to show any variation in the PoP of the ROS. Indeed, several is-mutant do not accumulate ROS, while the corresponding biological mutants were reported as sensitive to oxidative stress. However, under our simulation conditions many mutants (13/26 *in silico *mutants) showed an increase in the PoP of the ROS. This means that our model allows to point out the mutations the more prone to induce indogenous oxidative stress.

The "respiratory growth" phenotype reported in SGD is most often strictly related to the capability of the cells to grow on non-fermentative sources of carbon (glycerol, ethanol or lactate). All our simulations were run using glucose as the sole source of carbon. We should therefore not be able to evaluate the "respiratory growth" phenotype from our simulations. However, we show here that using the variations of the PoP of protons in the intermembrane space of mitochondria is indeed a very good is-marker of respiration defect. As much as 63/92 mutants descriptions could be validated this way, while 27/92 descriptions remained similar in is-mutants and is-WT and only 1/92 discrepancy was found between model and biology.

The "chemical compound accumulation" phenotype was very discriminating, with 33/45 descriptions in full agreement with the biology, 3/45 descriptions showing no change between is-mutant and is-WT. Our model did not allow to evidence accumulation of zinc in a is-zrt1 mutant, but this phenotype reported in SGD was conditional to the presence of aluminum ions in the growth medium of the biological mutant [44]. Some of the is-adk1 mutant phenotypes did not fit to the published ones, mostly because in this is-mutant, it is diffcult to quantitatively describe the imbalance between fermentation and respiration. The biological mutant exhibits apparently contradictory phenotypes, with increased ethanol accumulation and increased respiratory growth. Interestingly, our model predicts that the is-yfh1 mutant is depleted in oxidized glutathione, while SGD reports increased GSSG. However, recent results show indeed that the prediction from the model is correct [45] and this impacts on NADPH content due to higher G6PDH (Zwf1p) activity. All the mutants defective in "nitrogen sources utilization" were found in the simulations (5/5).

On a total number of 145 situations where the is-phenotypes were comparable to the SGD data, 91.7% were consistent (see Additional file 3). These results indicated that through a large scale analysis of the phenotypes expected from the biology, we were able to recover the corresponding is-phenotypes.

#### Analysis of the anaerobiosis to aerobiosis transition, or "how to deal with dynamic processes?"

**Figure 5:**
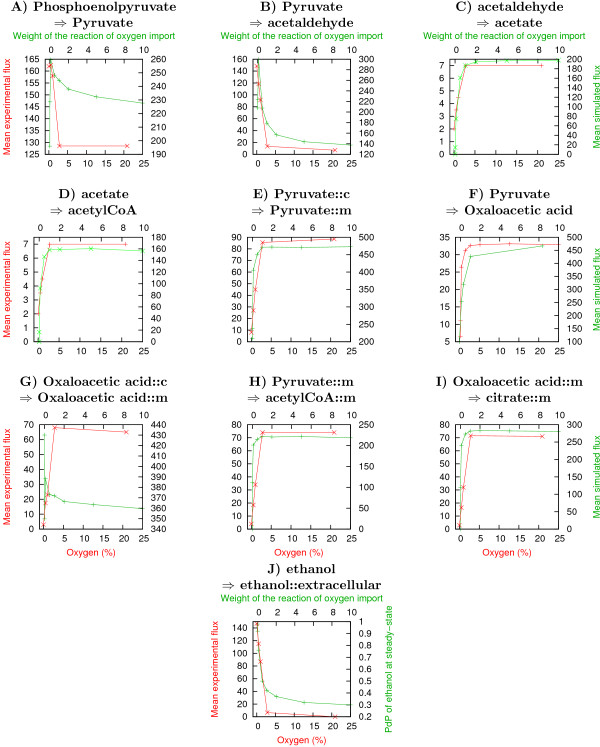
**Variations of the fluxes in selected reactions with and without oxygen**. Occurrence of 10 reactions (WT model) in the simulations, compared with experimental fluxes [36] (red). A-I: frequency of the reaction at steady state (green), J: PoP of ethanol at steady state (green).

We then analyzed the changes in several independent elements in the simulations of physiologically relevant transitions. One of our goals was to analyse the oxidative stress response. We therefore modeled the transition from anaerobiosis to aerobiosis. We analyzed simulations in which a key element, oxygen, was removed from the model. Oxygen is required to re-oxidise the reduced equivalents produced by cell metabolism. However, *S. cerevisiae *can grow in the absence of oxygen, due to its ability to shift from a respiratory to a fermentative metabolism. NADH is then oxidised by alcohol dehydrogenases, leading to ethanol production. We therefore compared the simulations of our model with different levels of oxygen to the experimental data of Jouhten *et al. *[36], who measured metabolic fluxes in yeast cells grown in a chemostat with a limited glucose supply in the presence of 20.9% oxygen (aerobic conditions) and under anaerobic conditions (0% oxygen). These experimental conditions were taken into account by weighting "glucose import" to a low value of 0.1. We compared the experimental fluxes with the reaction frequencies at steady state of key carbon metabolism reactions, in both the presence and absence of oxygen (Figure 5). All the *in silico *reactions displayed patterns of variation highly similar to those observed *in vivo*, with the exception of the reaction catalysed by Oac1p, a mitochondrial bidirectional oxaloacetate transporter with broad specificity for various anions. There is a simple explanation for the discrepancy observed for this specific reaction (of 10 considered). In anaerobiosis, cytoplasmic oxaloacetate is less produced than in aerobiosis. However, it is also less consumed than produced. Because the ratio of the production over the consumption is higher in anaerobiosis than in aerobiosis, oxaloacetate PoP is higher. Therefore, since one of the few remaining possible reactions involving oxaloacetate is transports by OAC1, the model predicts an increase in this transport in the absence of oxygen. Another important consideration when analysing results for the N_2_-O_2 _transition is that the model includes a number of regulatory mechanisms to describe the biological response of the yeast cell to oxygen deprivation accurately. These regulations were of two kinds. First, we modelled the induction, under anaerobic conditions, of the Rox1 responsive regulon [46,47]. Second, we modelled the effects of glucose repression on the alcohol dehydrogenase system, by adjusting the rate of degradation of the corresponding enzymes as a function of the presence or absence of oxygen [48]. This reflects the post-transcriptional regulation mechanisms involved in these processes.

This global validation of the model provides important evidence that the frequencies of utilization of the reactions during the simulations are qualitatively consistent with the metabolic fluxes observed experimentally.

We show here that our probabilistic Boolean modelling strategy may provide a useful description of the dynamics of biological systems. Further analysis is now required to determine the extent to which this key notion of fluxes, estimated as reaction frequencies at steady-state, can be generalised.

### Global analysis of the output of the simulations

**Figure 6:**
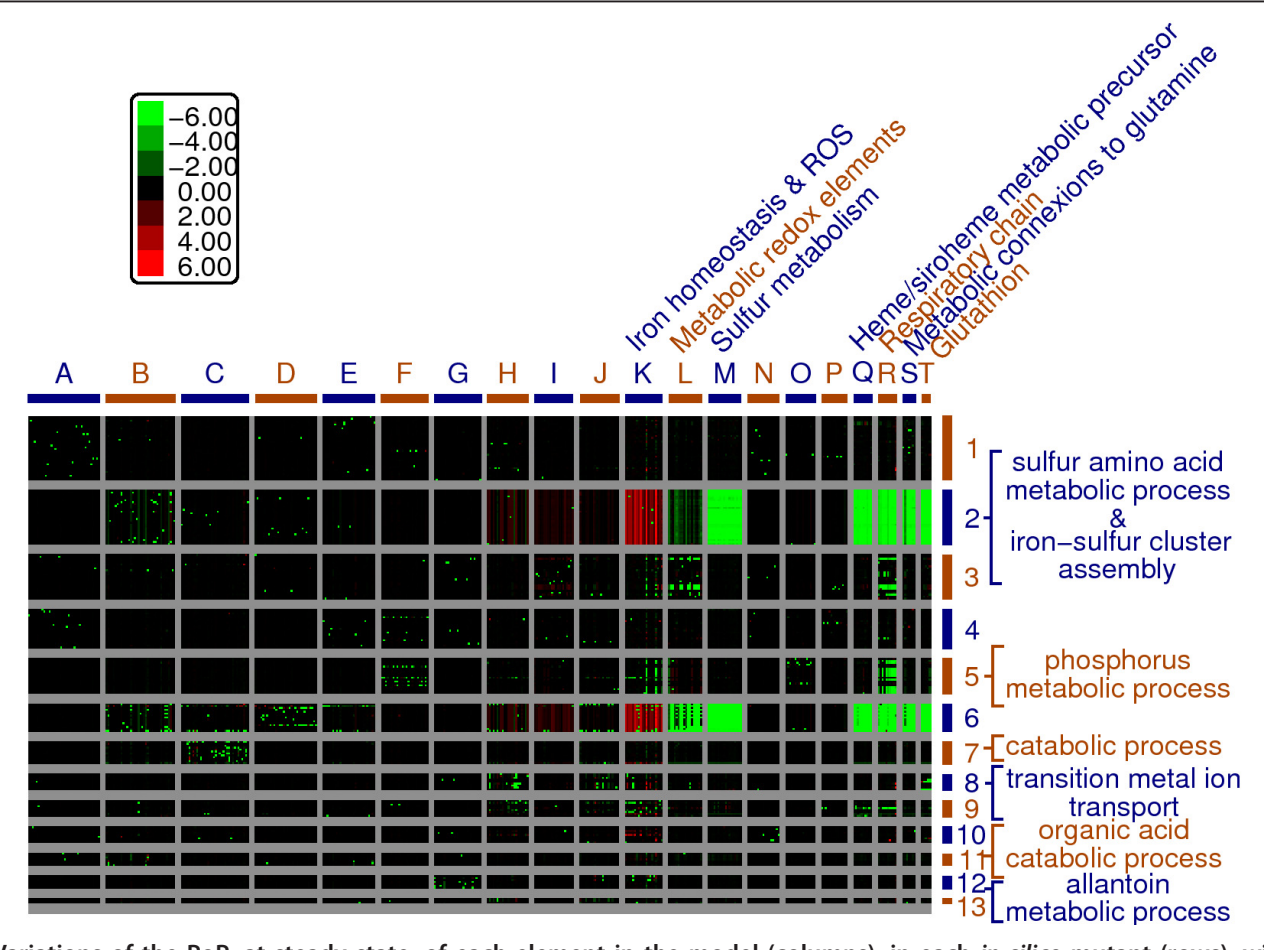
**Variations of the PoP, at steady state, of each element in the model (columns), in each *in silico *mutant (rows), with respect to WT simulations**. For each mutant and for each element,  was calculated. Positive values (red) indicate that the PoP was higher in the mutant simulations and negative values (green) imply that the PoP was higher in the WT simulations. For a complete content of all clusters see additional le 2 (cdt file of this clustering). See methods for details.

We evaluated the potential of the model to identify emerging properties through a global analysis of the simulations of the *in silico *mutants. *In silico *mutations were performed on 187 genes of the model. Indeed, it is impossible to predict the outputs of such a large number of simulations. We also included in this meta-analysis the simulations of models in which each of the 11 fixed elements was removed, independently, representing changes in the composition of the growth medium We evaluated whether it was possible to uncover from the simulations of the different models some specific properties of this complex system. A Bayesian classification algorithm (implemented at Autoclass@IJM, [49]) was then used to cluster the outputs of all simulations, defining classes of is-mutants with similar phenotypes and classes of elements varying in a similar manner in the simulations. Genes and elements were clustered into discrete sets (Figure 6 and Table 3). These mutations were grouped into seven classes that were consistent to some extent with the known biology of the system used (hem mutants, met mutants, cys mutants) and with additional mutations in genes with no straightforward relationship to the other members of the classes. This suggests that these mutations may define previously masked metabolic states, some of which it may be impossible to test experimentally, as several elements may be simultaneously missing, leading to cell death *in vivo*. A detailed analysis of each class showed that most simulations were correlated with biological data, but it was not always possible to predict the class to which a given gene would be assigned. Many is-mutations had only a limited impact on the steady-state PoP of most of the elements of the model when simulated with the predefined set of unlimited elements (Additional file 4). However, several classes of is-mutations had very pronounced phenotypes.

**Table 3:** Composition of the clusters of mutants from Fig. 6 (rows).

Cluster 1
PCK1 FBP1 POX1 IDH2 IDH1 PIC2 IDP2 GLN1 DUR3 MIR1 STR3 STR2 ALT1 NDI1 CTR2 MHT1 AAT1 GDH1 MSN5 ASN1 MAE1 LSC2 GGC1 DIC1 MDH3 ODC1 PHO89 MAC1 SOD1 ACS1 DUR12 CTP1 CYC7 AAC1
**Cluster 2**
CH3 MET14 MET16 ISU1 NFS1 ARH1 SUL1 NBP35 NAR1 ACO1 MET2 MET3 MET1 MET6 MET5 MET8 ATM1 HOMOSERINE KGD2 KGD1 CYS4 SAM1 MET25 CYS3 SERINE CFD1 YFH1 SO4 POS5

**Cluster 3**
TPI1 COX20 HEM14 COX1A13 SFC1 ZWF1 CTA1 PDX3 FAA2 SDH1 SDH3 SDH4 MDH1 NDE1 CCP1 COX2 HEM12 HEM13 HEM15 OSM1 CYC1 CYC3 CTT1 FUM1

**Cluster 4**
SAH1 PPN1 CDC19 PHO3 PHO2 PEP4 ROX1 LSC1 PHO91 ASP1 PYC1 PDA1 PHO12 COX5A COX5B SDH2 PHO81 CCC1 ADK2 MLS1 OAC1

**Cluster 5**
ATP689 CYT1 PHO84 ATP710A23 PHO4 QCR2 QCR6 QCR7 QCR8 COR1 PSE1 QCR9 PHO80 ATP1A5 PHO85 CYTB CYT2 QCR10 RIP1

**Cluster 6**
UTR1 O2 BUD16 COA HEM1 YAT2 ZN2 CRC1 HEM2 HEM3 HEM4 TPN1 CAT2 YIA6 ADK1

**Cluster 7**
HXK1 ALD2 ICL1 TDH1 FBA1 PFK2 PGK1 PFK1 ENO1 ENO2 GPM1 YNK1

**Cluster 8**
FET3 FTR1 ATX1 GPX1 GSH2 CU CCC2 CTR1 GSH1

**Cluster 9**
FET4 AFT12 ZAP1 ISA1 SSQ1 AFT1 AFT2 ZRT1 CIT1

**Cluster 10**
ADO1 PGI1 GLT1 SAL1 GRX3 MET22 GLR1 SOD2 SMF3

**Cluster 11**
ACH1 FOX2 LCCA POT1 ADH2 ADH1 PDC1

**Cluster 12**
FET5 DAL1 FTH1 DAL4 DAL3 DAL2 ALLANTOIN

**Cluster 13**
FE3 FRE1 CIT2

Each mutant is referred to by its gene name or the constant element that was turned "OFF". COX1A13 = genes COX1 to COX13; ATP1A5 = genes ATP1 to ATP5; ATP710A23 = genes ATP7 and ATP10 to ATP23; ATP689 = genes ATP6, ATP8 and ATP; LCCA = long chain carboxylic acid; ZN2 = Zn2+; FE3 = extracellular Fe3+; COA = coenzyme A.

One of these phenotypes was an increase in the PoP of toxic oxygen species. Two clusters of is-mutants displayed a large increase in the PoP of the hydroxyl radical. The first cluster (class 2) had defective sulphate assimilation, sulphur amino-acid synthesis and iron-sulphur cluster biogenesis or assembly. Among them were the sulphate permease (is-sul1), enzymes of the methionine/cysteine biosynthesis pathways (is-met1, 2, 3, 4, is-met8, 14, 16, 25) and enzymes involved in the mitochondrial assembly of iron-sulphur clusters (is-isu1, is-atm1 and is-yfh1, which is known to be extremely sensitive to oxidative stress). The cluster was particularly interesting, because it also grouped together is-mutations in genes and the depletion of elements involved in controlling major metabolic pathways (is-sam1, homoserine, CH3): CH3, representing the entry point to one-carbon metabolism, S-adenosyl methionine production (SAM1), and alpha-ketoglutarate dehydrogenase (KGD1 and KGD2), which connects carbon and nitrogen metabolism in yeast.

The second cluster (class 6) included is-mutants in which the early steps of haem synthesis (HEM1 to 4) were impaired, with phenotypes similar to wild-type models run in the absence of oxygen (except, of course, for the accumulation of ROS). A similar phenotype was observed for is-mutants with defective pyridoxal phosphate cofactor synthesis (PLP is required for ALA synthase activity). Interestingly, this class of is-mutants also included the WT minus Zn conditions, consistent with the requirement of Zn as a cofactor for ALA dehydratase (HEM2). The is-adk1 mutant, lacking adenylate kinase, also belonged to this class. All these is-mutants had a high PoP of toxic forms of oxygen. Most sulphur-containing compounds had a low PoP, due to the inability of these is-mutants to assimilate sulphate.

These two classes of mutations were strongly correlated with an iron-related phenotype, the accumulation in the nucleus of the iron-responsive transcription factor Aft1p, thus mimicking the "AFT1-up" phenotype described for biological Δyfh1 [50,51], Δatm1 [50] and Δcbf1 [52] mutants (Cbf1 is a global transcriptional regulator of the cys/met regulon [53]).

Is-mutants affected in the late steps of haem synthesis (HEM12 to 15, class 3) had very different phenotypes. They lacked cytochromes and displayed an increase in the PoP of hydrogen peroxide due to the lack of catalase, but their sulphur metabolism was unaffected.

These results show that our model is robust to *in silico *mutations, in that it predicts coherent phenotypes. To our knowledge, this is the first classification of such a large number of *in silico *mutations by a single model generating coherent and biologically relevant results.

Focusing on changes in the PoP of a given element makes it possible to explore multiple alterations to gene expression, such as loss of function (gene always "OFF") or overexpression (setting a higher weight). In this context, we might speculate as to which mutations might restore the wild-type level of a missing element in a given mutant, for example. Approaches of this type should help to target experimental studies trying to identify functional interactions between genes, but should also facilitate the exploration of new hypotheses relating to certain poorly documented aspects of the control of cellular homeostasis.

### Exploration of alternative hypothesis: an example of a use of the model

Although iron homeostasis in yeast is fairly well described, some aspects of its metabolism remain unclear. As an example, it has been reported that some mutant strains affected in the iron-sulphur biosynthesis pathway accumulate iron within mitochondria as iron-phosphate amorphous precipitates or nanoparticules ([54] (Δyfh1), [55] (Δatm1), [56] (Δyah1)). Iron-phosphate complexes (FePi) are virtually insoluble in aqueous media at physiological pH (Ksp 9.91 × 10^-16^) [57] and iron is therefore not available for biosynthesis. There should be some mechanism in wild-type cells that either prevent the formation of FePi complexes or allows the re-mobilization of iron for haem or iron-sulphur biosynthesis.

**Figure 7:**
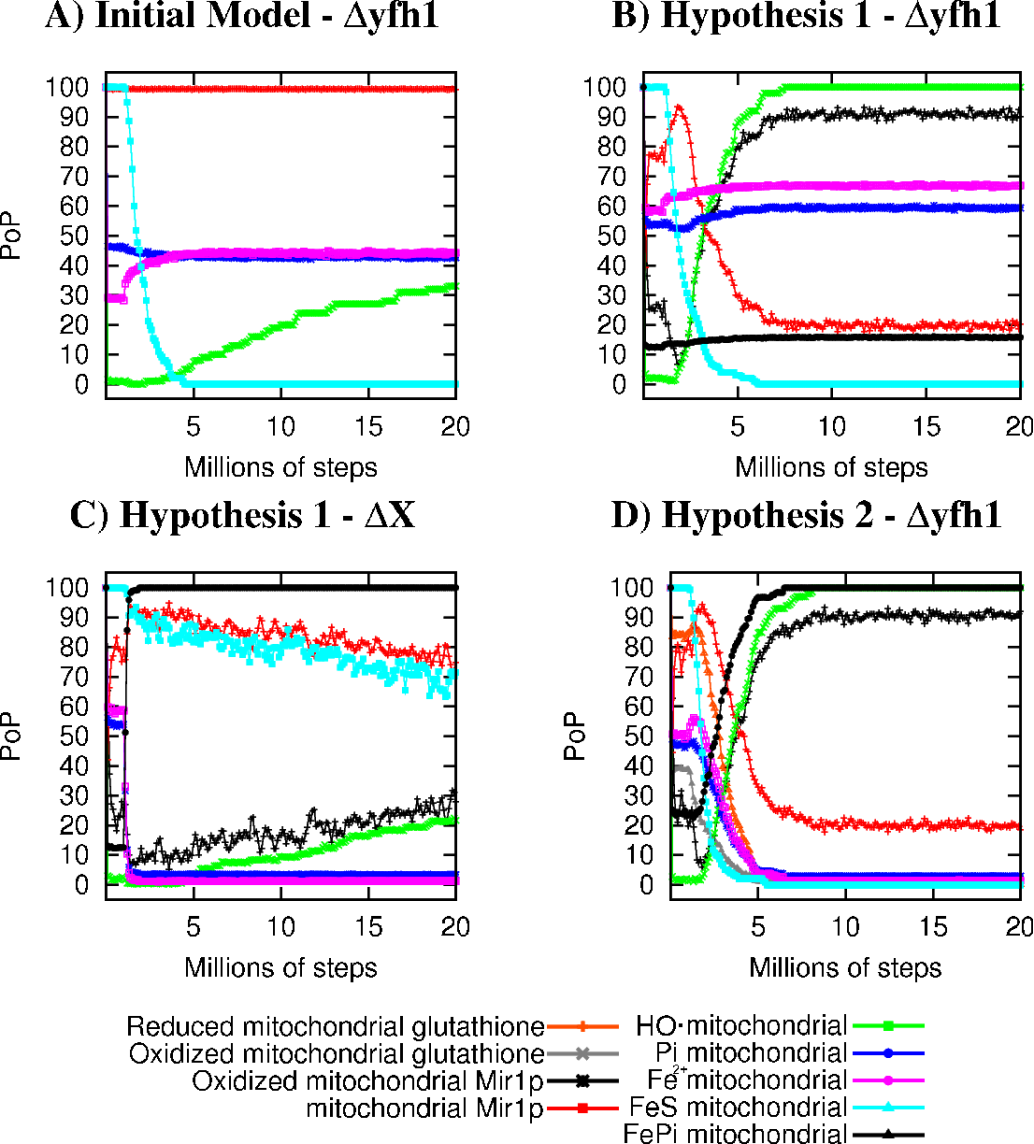
**Variation of selected elements PoP under different hyptothesis regarding the accumulation of iron-phosphate aggregates**. A: Initial Model - gene YFH1 set to "OFF", B: Hypothesis 1 - gene YFH1 set to "OFF", C: Hypothesis 1 - Element X set to "OFF". Values at steady-state: Oxydized Mir1p = 90%, non oxidized Mir1p = 20%, hydroxyl radical = 99%, FeS = 1%, D: Hypothesis 2 - gene YFH1 set to "OFF".

We used our model to explore alternative hypothesis related to FePi accumulation within wild-type and iron suphur deficient cells (the Δyfh1 mutant as an example). We first postulated the presence within cells of a highly efficient reaction that reverts FePi aggregates to free Fe and Pi. This reaction is catalyzed in the model by an unknown element "X" and the weight of its reaction is set to 1000. As shown in Figure 7-C, removing this reaction from the model led to a dramatic increase in the PoP of mitochondrial FePi, a decrease of both mitochondrial Fe and Pi and a steadily increasing oxidative stress concomitant to the loss of iron-sulphur clusters.

We wanted however to evaluate the evolution of the model in situations where the unknown element "X" was present, using different hypothesis of physiological relevance. The first hypothesis takes into account the well described alteration of the function of the major Pi transporter, Mir1p, when a single cysteine residue is modified (see Additional file 5). Mir1p shifts from an anti-port (Pi/Pi or Pi/OH^-^) activity to a symport (Pi/H^+^) activity [58]. We therefore simulated this change of activity when Mir1p is the target of an oxidative modification under stress conditions. Figure 7-B shows that while the wild-type cells are unaffected by this modification, a yfh1 mutant, known to be very sensitive to oxidative stress and to accumulate FePi, indeed exhibits a slight but significative increase in Fe, Pi and FePi PoP within mitochondria, while ROS PoP strongly increase and FeS dramatically drops.

However, in the model, Mir1p is not the only transporter involved in the exchange of Pi between the cytoplasm and the mitochondria. Sal1p (ATP-ADP/Pi antiporter) and Dic1p (dicarboxylic acids/Pi antiporter) also contribute to the Pi homeostasis. We therefore simulated a model where all the three transporters may have their activities altered by an oxidative stress. The phenotypes were similar to those observed under the previous hypothesis but the PoP of FePi was only slightly higher (data not shown), indicating a modest cumulative effect of the oxidative alterations of the three transporters.

FePi nanoparticles are dissociated *in vitro *when treated with hydrosul fite, a strong reductant [56]. We evaluated the evolution of the model when "X" was identified as a thiol as a potential reducing agent. In our model, this thiol may be either cysteine or reduced glutathione. Removing cysteine impacts dramatically on the model and precludes the evaluation of its role in FePi formation. Therefore, we tested the hypothesis that "X" may be glutathione. Figure 7-D shows that under this hypothesis, most of the phenotypes of the biological yfh1 mutant are appropriatly described in the model since in this mutant, the glutathione PoP is 0% at steady state: there is a strong increase of the PoP of FePi in addition to the previously observed high PoP of ROS, loss of iron-sulphur clusters and drop in mitochondrial glutathione. This example shows how the model may be used to investigate poorly understood aspects of the cell biology. Differents hypothesis were explored to evaluate the mechanism of the accumulation of iron-phosphate within mitochondria. The simulations indicated that, although perturbation of phosphate transport accross the membrane could be considered, the hypothesis of a reductive dissociation of FePi by thiols is more likely. Indeed, many of the phenotypes of the mutant simulated are adequately rendered in this latter case. We are currently trying to test this hypothesis by using combination of biological mutants such as the Δ*gsh1*. However, this study is made difficult since a Δ*gsh1 *strain was shown to have limited growth without exogeneous glutathione. The lack of glutathione triggers an apoptotic response after few cell divisions [59]. The Δ*gsh1 *strain accumulated mitochondrial iron but the activity of some FeS enzymes within mitochondria remained normal (the cytosolic FeS pool was deeply depleted) [60]. Interestingly, the double mutant Δ*gsh1 *Δ*atm1 *was described as non-viable.

The example of the investigation of the mechanisms of accumulation of FePi within mitochondria in pathological situations illustrates the power of this approach. Several hypotheses are easily explored by introducing the appropriate reactions and elements into the initial model. Testing different hypotheses paves the way to different type of experiments such as genetic/biochemical identification of the "X" element that makes iron soluble even in the presence of phosphate, with special emphasis on glutathione or other thiol containing compounds status within the cells. It will be also necessary to measure the activity of the various phosphate exchangers in mitochondria purified from different mutant strains, or from mitochondria subjected to strong oxidative stress. We believe these applications of the model will contribute to a better understanding on what contribute most to the control of iron homeostasis. This approach is to be extended to the analysis of other pathways of important physiological relevance, in normal or pathological cells.

### Application of this methodology to other systems

The new extension of the Biocham language introduced and validated here could potentially be extended to many other studies. Systems biology papers frequently contain "interaction maps" for cellular or biochemical processes, as defined in a number of studies (see for example [16,61]) based on the formalism of Molecular Interaction Maps http://discover.nci.nih.gov/mim. Several authors have suggested that their interaction maps could serve as a first step towards the development of a computational model and have highlighted the need for appropriate tools to translate these maps into models [62]. Our approach appears highly suitable for this purpose. Each edge of an interaction map could be translated into a rule, as used in this paper. The weights of rules may be defined as a function of biological knowledge, as shown here. This last step seems to be manageable, because these maps are generated by qualified biologists and are based on extensive mining of published texts. The definition of weights simply provides a means of translating a biologist's intuition and knowledge into a numerical value; this value does not need to be obtained through experimentation (although it could be) and requires definition only with respect to the other rules. Our sensitivity analysis shows that only the orders of magnitude for the weights are relevant. Biologists should then simulate and analyse their models. We believe that the analysis of simulations will prove to be a powerful tool for the analysis of large biological systems [63]).

Such a qualitative approach may be seen as a first step towards more detailed mathematical models. Indeed, no quantitative value is required and only a rough description for each interaction is needed. Despite providing only an approximation of the interaction maps, we believe that this method can provide biologists with meaningful information.

## Conclusions

Iron homeostasis, oxidative stress and metabolism are tightly linked within cells, with far-reaching implications at the level of the whole organisms. Indeed, disturbances in these processes are increasingly frequently being linked with disease. However, it remains unclear how the disruption of part of this system affects the rest of the network. Previous studies have modelled iron-related processes at the subcellular level (iron-sulphur cluster assembly in yeast mitochondria [64,65]), at the cellular level in *E. coli *[33] (modelling of the genetic regulation of iron-related genes) or at the organism level in humans (regulation of iron levels; [30,34,35,62]. Other models dealing with oxidative stress-related systems have been published [66,67] but, to our knowledge, this is the first large model to include iron homeostasis as well as oxidative stress and some aspects of metabolism.

Using a Boolean probabilistic methodology based on the rule-based approach proposed by Biocham, we have constructed a detailed model of this complex system in yeast. Our methodology uses:

• Only positive statements (no OR or NOT) to reflect the molecular nature of biological interactions,

• Weighted reactions to reproduce large differences in certain reaction rates,

• Stochastic simulations, to reproduce the variability of biological systems.

This new methodology combines the adaptability of Boolean models (no need for quantitative data for every element or reaction, possibility of including "fuzzy knowledge" etc.) with the need to reflect the reality of biological processes as closely as possible.

The resulting model consists of 642 elements and 1007 reactions, including iron homeostasis, oxygen-related reactions and the main carbon, nitrogen, sulphur and phosphate pathways. It includes several levels of regulation: gene expression ("housekeeping" or conditionally expressed genes), proteins production, protein targeting to specific subcellular locations and degradation, nutrient supply and transformation through the main metabolic pathways and the uptake and use of ions. Combined with our simulation algorithm, this model can be used to investigate the effects of specific perturbations on simulations, facilitating direct comparison with the "wild-type" situation. These perturbations may be changes in the "growth conditions" or gene deletions.

The analysis of simulations for selected *in silico *mutants showed the consistency of the model to specific experimental phenotypes. Comparison of the simulations of the wild-type model run with and without oxygen to experimental metabolic fluxes measured under similar conditions showed that most aspects of the adaptive responses of the system were accurately addressed. In a global analysis, *in silico *mutations generated for each gene of the model were compared with the "wild-type" model. A clustering analysis for all the simulations led to the identification of clear phenotypic profiles, thus providing new insights into some metabolic response to stress conditions. Finally, the model was also used to explore several new hypotheses in order to better understand some unexpected phenotypes in given mutants. All these results show that this model, and the underlying modelling strategy, are powerful tools for improving our understanding of iron homeostasis in relation to the main features of metabolism, energy production and oxidative stress.

## Methods

### Building the model

#### The list of reactions

As a starting point, the *S. cerevisiae *bibliome was searched for references related to a list of genes known to be involved in iron homeostasis. The bibliome consist of all articles concerning *S. cerevisiae *found on PubMed.

From these articles, a list of reactions was manually inferred and selected on the basis of our knowledge.

The new genes cited in these articles that we felt to be critical for model accuracy were then used to direct a new PubMed search. This process was repeated until we were unable to identify any new reactions strictly related to iron homeostasis.

The same process was used for inorganic phosphate homeostasis, for some oxidative stress reactions and for any other processes that we felt to be important for our model that were not described in databases.

We searched the Swissprot database for a list of proteins requiring iron-sulphur clusters or haem as a cofactor. We then selected metabolic pathways involving these proteins that could be directly or indirectly linked to iron homeostasis or oxidative stress. The reactions describing these pathways were expressed according to SGD pathways [68] on the *Saccharomyces *Genome Database website http://www.yeastgenome.org.

However, when including a given pathway, we did not systematically describe all its steps. If a pathway included several steps producing intermediate metabolites not required by any other pathway included in our model, we wrote the whole pathway as one reaction, unless we had reasons to include the intermediate reactions. For example, siroheme biosynthesis from uroporphyrinogen-III involves three reactions. The two intermediate metabolites produced, precorrin-2 and sirohydrochlorin, are not required by any other reaction already included in the model, so we could have expressed the whole process as one reaction. However, the first step involves S-adenosyl-methionine and S-adenosyl-homocysteine, which are already included in several reactions in our model, and the last step involves iron. Thus, if siroheme cannot be produced - in a mutant for instance - we want to be able to determine whether this deficiency is related to 2-S-adenosyl-methionine synthesis or iron availability. We therefore included siroheme biosynthesis as two reactions, the first producing precorrin-2 from uroporphyrinogen-III and 2-S-adenosyl-methionine and the second producing siroheme from precorrin-2 and NAD.

Finally, we searched the yeast metabolome (described in SGD pathways) for reactions that might link several metabolites already included in our model. This is the reason for which we included alanine degradation, for example, in the model.

#### The weights of the reactions

The default value for the weight of the reactions was one. However some reactions were given a weight lower than one (for most degradation reactions, the weight is 0.01), or higher than one. For example, the reaction catalysed by the Sod1 superoxide dismutase was given a weight of 100, to take into account the extremely high abundance of this protein in yeast cells (519,000 molecules per cell; [69]), its very high catalytic efficiency and turnover number. A list of reactions given weights other than one is provided in Table 2. We simulated our model with all weights set to 1, and it was unable to produce realistic outputs (data not shown). For example, the hydroxyl radical elements have PoP lower than 1% in the complete model, whereas the PoP is higher than 70% in the model without weights, which is not realistic (the parameter PoP is defined below, in the Simulations subsection). The WT model corresponds to a model where the outputs are biologically meaningfull according the data of the literature and our own experience. We performed a sensitivity analysis of the outputs of the model (PoP at steady state) when the weights of the reactions are modified. Each weight was multiplied by a coefficient k. The differences between each PoP of the initial model and the PoP of the model with the modified weight were computed. Our results show that the model is robust to modifications of the weights when the coefficient k ranges from 0.1 to 10. See Additional file 2 for more details.

### Simulations

#### Algorithm

We used a modified version of the Biocham asynchronous Boolean simulation algorithm, which can be summarised as follows:

1. Initial state: the list of elements that are "ON" at the beginning of the simulation.

2. Based on the list of elements that are "ON", the list of possible reactions is inferred: A reaction is possible if its reactants and modifiers are "ON".

3. A reaction is randomly selected from the list of possible reactions

4. The products of the selected reaction are set to "ON".

5. The reactants are randomly either set to "OFF" or left "ON".

6. A new list of elements that are "ON" is computed.

7. Steps 2 to 6 are repeated for each simulation step.

In the Biocham algorithm, the reactants are not always set to "OFF", so it is possible to reselect the reaction in subsequent steps (see above step 5). This possibility reflects the presence of more than one molecule of each sort in biological systems. If the same reaction is selected over and over again, all the reactants will eventually be turned "OFF" and the reaction will cease to be possible unless other reactions set the reactants back to "ON". Indeed, in biological systems, all elements may be considered to exist in limited quantities.

We also had to overcome a limitation of Biocham to obtain simulations as close as possible to real biological systems: this algorithm does not mimic the large differences in reactions rates observed in real biological systems. Although we do not precisely know the rate of all the reactions in the model, we can reasonably state that the degradation of an enzyme is far less likely to occur than the reaction this enzyme catalyzes. Another example is related to the functioning of the TCA cycle: we know that some reactions of the cycle are technically reversible but the reaction always runs in one direction in practice, because this direction is thermodynamically more favorable. We needed to mimic this situation, to make our model as realistic as possible. We therefore decided to extend the algorithm, by weighting the reactions. From the weights of the possible reaction, at step 2, in the previous algorithm, we calculated the probability of a reaction being selected as the weight of the reaction divided by the sum of the weights of all possible reactions. A reaction is therefore more likely to occur if it has a high weighting.

If the experimental rates of all reactions in the model were known, we could set their weights accordingly. As these rates were not all known, we defined relative weights, using a default rate of one. The weight of a given reaction was modified if we had quantitative or qualitative experimental knowledge relating to its reaction rate (see Table 2 for a list of reactions with weights other than one).

In this paper, all the elements were set to "ON" at the beginning of the simulations, defining thus the "initial conditions". We tested different initial conditions (for the non constant elements only) which always resulted in the same steady-state (data not shown).

#### Mean profiles

The simulation algorithm was developed in the C (for computation) and Python (for file/data management) languages on a Linux workstation. The simulations were performed on a cluster of 40 nodes (DualCore AMD 64 bits Opteron bi-Processor, 2Go RAM, PBS/Maui scheduler). Each type of model was simulated 100 times, until a steady state was reached (see below). From these 100 simulations and for each element, we defined the PoP as the percentage of simulations in which the element was "ON" (present), for each simulation step. This calculation process is referred to as the profile of an element. Figure 4 shows examples of such profiles in which the mean PoP was also calculated every 100,000 steps.

#### Steady states

We checked whether steady state had been reached, by performing an ANOVA on the last 2 million steps, for every element (the null hypothesis is that the PoPs are independent from the iterations). If a significant result was obtained (p-value below 0.001), then the simulation was rerun for a larger number of steps. As background noise in the simulation can generate false positives, if the means of the last million steps of the new simulation of each element was equal to the means of the previous simulations, then we considered steady state to have been reached.

For some mutants, some elements have PoPs that increase or decrease slowly. For example, in some mutants the reactive oxygen species have increasing PoP. Most of them will have a PoP of 100 at steady-state but some will reached this maximum value faster than others. We advocate that comparing the simulations of the mutants after the same number of steps may be related to some biological properties of the mutants. To be able to take into consideration these differences in our systematic *in silico *mutants analysis, we used the PoP of the elements after 20 millions of steps even though the simulations were not at steady-state yet.

74% of the simulations reached steady-state before 20 million steps and among the remaining 26% only some very slowly increasing or decreasing elements have not reached steady-state yet.

#### In silico mutations and mutants

Model simulations began with all elements "ON" and continued until steady state was reached: this situation corresponds to "wild-type" simulations. To simulate a mutant for a given gene, a wild-type simulation was carried out for one million of steps. The gene in question was then turned "OFF" and the simulation was continued for 19 more millions of steps for the systematic mutants analysis or until steady-state was reached for the other analysis. This method for simulating mutants mimics experimental Tet-OFF mutants, in which the transcription of a given gene is controlled by a tetracycline- responsive promoter and can be turned off by adding tetracycline to the growth medium [70].

The model was simulated according to this procedure with, for each set of simulations, one of the unlimited elements deleted. Each type of model in which an unlimited element was turned "OFF" was referred to as a "mutant". Note that none of the reactions were modified.

#### Clustering

For each "mutant" model, the mean of the last million of steps of the simulation was calculated for each element. Then, for each element and for each "mutant", a distance to the "wild-type" was calculated as follows:  (formula based on A.Ultsch RelDi [71]). In the resulting matrix, each column is an element of the model and each row a "mutant". This matrix was then clustered, using Autoclass@IJM (real location data type, relative error = 0.01), which identifies classes of "mutants" causing similar changes in the simulations. The matrix was then transposed and clustered again: this generated classes of elements changing in a similar fashion in the different "mutants". Figure 6 shows the matrix clustered for both elements and "mutants".

Figure 6 is annotated: the rows are annotated with the most significantly enriched gene ontology process (calculated with GoTermFinder [72]), the columns were annotated manually (only the columns containing many elements which PoP changes significantly were annotated - see Additional file 6 for the complete clustering).

#### Phenotypes analysis

The file containing the manually curated phenotypes of the yeast mutants was retrieved from SGD (file edited the 03/13/10). From this file, we extracted the phenotypes associated with mutants of genes present in the model. From this selection, we further extracted the phenotypes that could be compared with our simulation results (e.g. the auxotrophies related to molecules present in the model). Six types of phenotypes were extracted: "auxotrophies" (cysteine, methionine, heme, glutamate), "chemical compound accumulation" (of elements present in the model), "oxidative stress resistance", "respiratory growth" and "nitrogen source". Then, we compared these phenotypes with the PoP of the corresponding elements in the WT model and in the mutants. For the "oxidative stress resistance", we did not simulate the mutants with an additional source of stress, as where observed the experimental phenotypes. Instead, we looked for the production of ROS in the mutant as compared to the WT in standard simulations. Therefore, only the constitutively stressed mutants showed similar phenotypes in our simulations and *in vivo*. As for the "respiratory growth" phenotypes, we compared the PoP of the protons in the intermembrane space (noted "Hinter::mitochondria" in the model), because in the model an increase or a decrease in this element PoP can be directly linked to a defect in the respiratory metabolism. For the "nitrogen source utilization" phenotypes, we compared the PoP of the products of the utilization of the nitrogen source. See Additional file 3 for the full results.

## Authors' contributions

FA and JMC built and validated the model. FA and DM implemented the simulator and performed the simulations. FA created Tables and Figures. FA, JMC and DM wrote the manuscript. All authors read and approved the final version of the manuscript.

## Supplementary Material

Additional file 1 **The WT model as used for the simulations**. The WT model as used for the simulations. Lines beginning with "%"are comments. Each reaction of the model is followed by its weight (separated by a tab spacing). Elements with name beginning with "#"are constant elements. The cellular location of an element is separated from the element name by "::". Reactions may take four main forms: 1) *A *+ *C *= >*B *+ *C *(A gives B using C) 2) *A *= [*C*] = > B (different formalism for the same reaction) 3) A = > (A is degraded) 4) A < = > B (reversible reaction). Click here for file

Additional file 2 **Sensitivity analysis of the outputs of the model (PoP at steady state) when the weights of the reactions are modified**. Click here for file

Additional file 3 **PoP of elements in simulated mutants versus phenotypes manually curated by SGD in the corresponding mutants**. The spreadsheet le is composed of 6 sheets (one for each type of phenotype). Each sheet contains the simulated PoP on the left and the SGD phenotypes on the right panel (as they appeared in the original file). One phenotype can be represented by multiple lines when multiple articles reproduced it. ND = no difference between the WT and the mutant were observed. Red cells mean that the differences observed in the *in silico *mutant is not in agreement with the experimental observations. Yellow cells mean that the differences observed in the *in silico *mutant is not in agreement with the experimental observations as referenced by SGD but that a more recent publication is in agreement with our simulations (the happened once, see text for more details). Columns in the "SGD Reference" section: - Reference (SGD REF Required, PMID optional) -PMID: #### SGD REF: #### (separated by pipe)(one reference per row) - Experiment Type (Mandatory) -The method used to detect and analyze the phenotype - Mutant Type (Mandatory) -Description of the impact of the mutation on activity of the gene product - Allele (Optional) -Allele name and description, if applicable - Strain Background (Optional) -Genetic background in which the phenotype was analyzed - Phenotype (Mandatory) -The feature observed and the direction of change relative to wild type - Chemical (Optional) -Any chemicals relevant to the phenotype - Condition (Optional) -Condition under which the phenotype was observed - Details (Optional) -Details about the phenotype - Reporter (Optional) -The protein(s) or RNA(s) used in an experiment to track a process Click here for file

Additional file 4 **List of the elements of the model that are always "ON" during the simulations**. Click here for file

Additional file 5 **The WT model implementing the first hypothesis**. The WT model implementing the first hypothesis explored in "Results/Exploration of alternative hypothesis: an example of a use of the model". Click here for file

Additional file 6 **Clustering presented in Figure **6. cdt file (readable using Java TreeView or a spreadsheet program) of the clustering presented in Figure 6. Click here for file
