# Supplementary material for: A Boolean probabilistic model of metabolic adaptation to oxygen in relation to iron homeostasis and oxidative stress
Source: BMC Syst Biol. 2011 Apr 13;5:51. doi: 10.1186/1752-0509-5-51 (PMC3094212; doi:10.1186/1752-0509-5-51)

Achcar et al. "A Boolean probabilistic model of metabolic adaptation to oxygen in relation to iron homeostasis and oxidative stress"

Sensitivity analysis of the model.

The weights of the reactions in the model are either set with a default value or derived from the literature. At which extent are the PoP of the elements influenced by the weights of the reactions ? To answer this important question, we performed a sensitivity analysis. The weights of the reactions used in the paper are referred to as "the reference weights" in the following. These weights range from 0.001 to 100 (see table 2 in the text).

The model comprises 1007 reactions, some of them being bi-directional, so there is a total of 1029 uni-directional reactions to test. In order to perform the sensitivity analysis, we multiplied the 1029 weights, one by one, by a coefficient *k*, and ran simulations until steady state was reached, using the same strategy as described in the text (see section "Methods"). We selected the following values for the coefficient *k* : 0.1, 0.2, 0.5, 0.9, 1.1, 1.2, 1.5, 5.0, 10.0.

From these simulations, we computed *k* sensitivity matrix *S(k)*, where *Sij(k)* represents the difference between the PoP of the *i*-th element when the model if simulated with reference weights and the PoP of the same element when the model is simulated with the weight of the *j*-th reaction multiplied by *k*. Thus, each *S(k)* matrix has 1029 rows (one for each reaction) and 430 columns (one for each non-constant element).

In order to synthesize this bulk of information, we defined a threshold to assert that a *Sij(k)* value is "significant" or not. Here, "significant" means that the PoP of the *i*-th element is different when the weight of the *j*-th reaction is multiplied by k (compared to the PoP obtained with the reference weight). We choose a threshold value of 5%. Recall that values of PoP range from 0% to 100%, so we assessed that an absolute difference less than 5% should not be considered as significant.

For a given value of *k*, and for each reaction, we then computed K, the number of elements that have a significant *Sj(k)*. The smallest this value, the less sensitive the model is to the modification of the weight of the *j*-th reaction.

Then, we reported in an histogram the number of reactions that have K=0 significant *Sij(k)* (this case correspond to non-sensitive reactions, i.e. the modification of these reactions by a coefficient *k* do not change the PoP of the elements compared to the reference model), then the number of reactions that have K ϵ [1, 5], K ϵ [6 ,10], K ϵ [11,15] and K ϵ [16, 20]. See Figure below.

It is worth noting that for coefficients *k* = 0.9 (decrease of 10% of the value of the weight) and *k* = 1.1 (increase of 10% of the value of the weight), no PoP are modified compared to the reference model.

A higher number of significant values of *S(k)* was observed when we decreased the weights of the reactions by 50% of their value (*k* = 0.5) : 640 modified reactions over the 1029 do not significantly change the PoP of any element; 138 modified reactions lead to changes of 1 to 5 values of PoP, and 6 modified reactions lead to changes of 6 to 10 values of PoP. We made similar observations when each weight of a reaction was increased by 50% of its value (*k* = 1.5) : 709 modified reactions do not influence the values of any PoP and 39 modified reactions have an impact on 1 to 5 PoP values.

Analyzing which values of PoP, and therefore which element, are sensitive to which reaction showed that the most sensitive elements are the products of the modified reaction as expected. Our results show that varying the weight of a reaction from 0.1 to 10 times its initial value has a small influence on the overall model. This demonstrates that the model is robust and that each modification has only a local impact on the graph (as defined in figure 2 in the text).

As a conclusion, the sensitivity analysis of our model showed that our model is robust to

modifications of the weights up to their multiplication or division by 10, using a large number of simulations (1029 weights, 9 coefficients per weight).

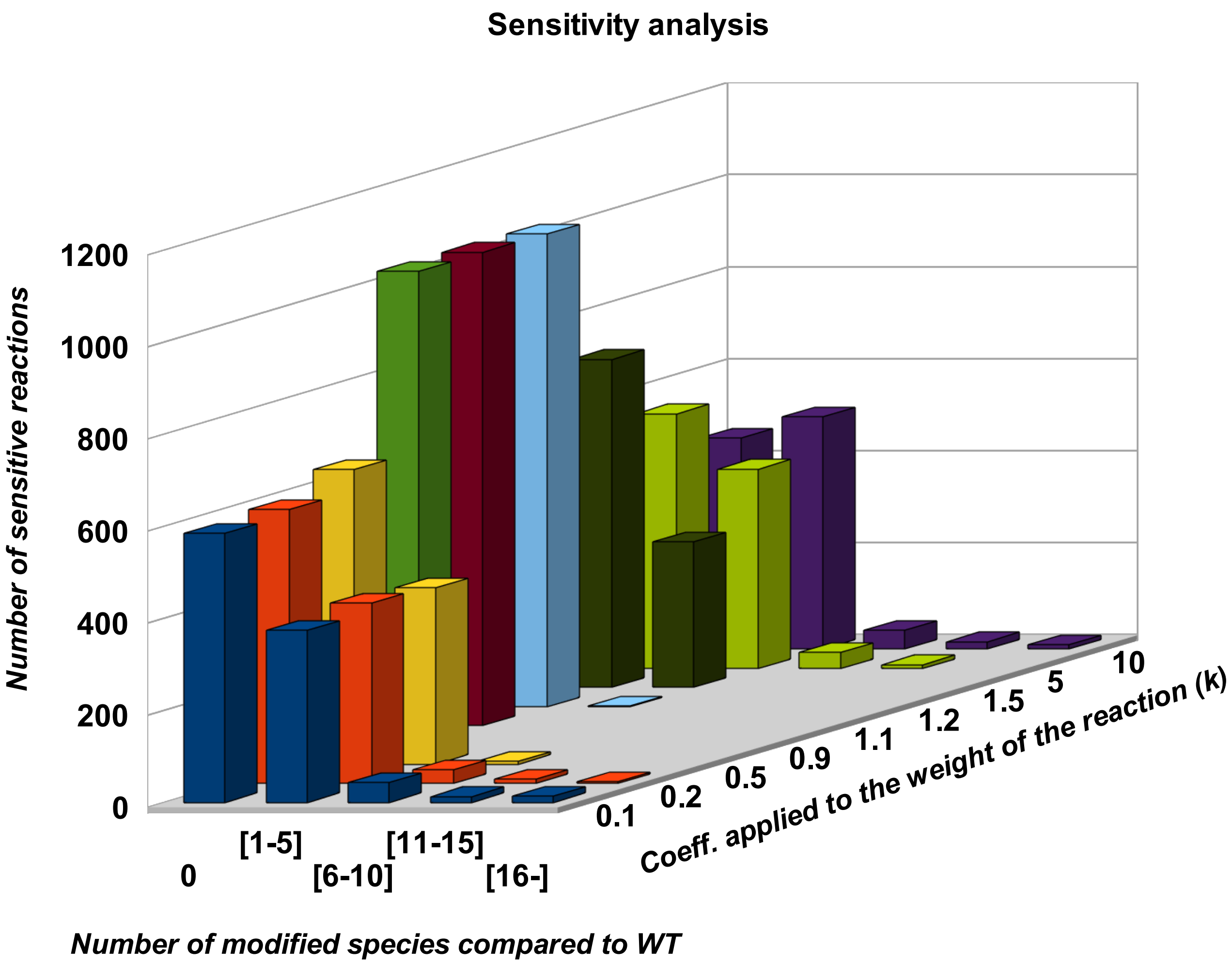

Supplement: Additional file 2 — Sensitivity analysis of the outputs of the model (PoP at steady state) when the weights of the reactions are modified. [file 1752-0509-5-51-S2.PDF]
